# Unravelling the decision making of foraging vultures: insights from a field experiment

**DOI:** 10.1098/rsos.250085

**Published:** 2025-04-16

**Authors:** Eneko Arrondo, Jorge Martinez Carracedo, Patrick McAllister, Zebensui Morales-Reyes, Martina Scacco, Roberto Pascual-Rico, Ainara Cortés-Avizanda, José Antonio Donázar, Marcos Moleón, José Antonio Sánchez-Zapata

**Affiliations:** ^1^ Estación Biológica de Doñana (EBD), CSIC, Sevilla, Spain; ^2^ University of Ulster, Coleraine, Northern Ireland, UK; ^3^ Departamento de Biología Animal, Edafología y Geología, Facultad de Ciencias, Universidad de La Laguna (ULL), San Cristóbal de La Laguna, Spain; ^4^ Department of Migration and Immuno-Ecology, Max-Planck-Institut fur Ornithologie, Radolfzell, Germany; ^5^ Department of Biology, University of Konstanz, Konstanz, Germany; ^6^ Research Institute of Hunting Resources (IREC), CSIC, Ciudad Real, Spain; ^7^ Instituto Mediterráneo de Estudios Avanzados (IMEDEA), CSIC, Esporles, Illes Balears, Spain; ^8^ Conservation Biology, Consejo Superior de Investigaciones Cientificas, Madrid, Community of Madrid, Spain; ^9^ Department of Zoology, University of Granada, Granada, Spain; ^10^ Department of Applied Biology, Miguel Hernández University of Elche, Elche, Spain

**Keywords:** accelerometer, GPS tracking, griffon vulture, *Gyps fulvus*, optimal foraging theory, type I functional response

## Abstract

Optimal Foraging Theory (OFT) integrates both the consumer and the resource, yet their simultaneous assessment is uncommon. Vultures represent an ideal model for OFT studies because carrion requires no capture effort and minimal handling, allowing them to focus primarily on food searching. Here, we combined GPS tracking of 61 Iberian griffon vultures (consumers) with photo-trapping monitoring of 49 carcasses (resources) to assess the determinants of vulture foraging and the consequences for carrion consumption in two areas with different carrion abundances. First, we determined the importance of different factors (distance to the resource, hunger and competition) in the decisions of individuals of whether to descend or not on a carcass. Second, we compared carrion consumption patterns (time of carcass discovery and consumption, and maximum number of vultures gathered around the carcass) between areas. We found that distance, rather than hunger, is the primary factor determining whether a vulture descends to a carcass. In parallel, carrion was consumed similarly in areas with different resource availabilities. These findings indicate that vultures tend to eat whenever a nearby opportunity arises, consistent with a type-I functional response.

## 1. Introduction

Since the pioneering study by MacArthur and Pianka in 1966 [1], the Optimal Foraging Theory (OFT) has become essential in understanding which resources a consumer should choose and the strategies they use to do it. OFT posits that foragers aim to maximize their energy intake during foraging activities [1–4]. This framework not only helps explain consumer behaviour, but also provides insights into the dynamics of resource consumption. From the perspective of consumers, innumerable studies have examined how intrinsic and extrinsic factors influence the balance between the costs and benefits of foraging. Studies focusing on benefits have highlighted factors that enhance the probability of finding food, such as the use of social information [5]. Conversely, costs are generally divided into two key stages of foraging: the *searching* phase, encompassing the energy and effort invested in locating resources [6], and the *handling* phase, which involves the capture, preparation and consumption of the prey [7,8]. From the resource perspective, individual foraging decisions ultimately determine how quickly and completely a resource is consumed or how many consumers feed on it, impacting broader ecological structures and functions [9]. Regardless of the significance of these aspects, applying OFT principles to simultaneously assess foraging decisions and their impact on resource consumption patterns is scarce in the scientific literature.

Despite the existence of numerous theoretical models of OFT, empirical investigations are considerably more constrained due to the challenges in disentangling the factors and relative energetic costs associated with each stage of foraging, i.e. searching and handling. Different resources often present distinct characteristics in terms of abundance, spatiotemporal distribution and accessibility, which complicates obtaining integrative assessments [10]. A possible strategy to simplify research within the consumer-resource framework is to focus on species with extreme foraging behaviours, i.e. in which one of the two foraging phases is either absent or significantly reduced. Trapping spiders, for instance, exemplify species without a distinct searching phase; they rely on webs to catch prey and thus allocate most of their energy to constructing and maintaining these webs, which is part of the handling phase [11]. However, the opposite case, namely species where the handling phase is minimal, has received far less attention. This is in part because searching usually involves movement, which requires challenging monitoring schemes. Prior to the development of advanced biologging systems, methods for tracking large animal movements were laborious and often required expensive equipment, such as gliders or light aircraft [12]. The advent of modern tracking technology has dramatically improved the ability to study animal movement in detail, providing new opportunities to explore and understand the dynamics of foraging behaviours.

A paradigmatic group of animals with minimal handling effort are vultures, which are the only obligate scavengers among terrestrial vertebrates [13]. Unlike predators that hunt live prey, requiring significant handling to capture their prey [8], vultures primarily focus their foraging efforts on searching for dead animal carcasses [13]. Carrion is an ephemeral and relatively unpredictable resource with minimal handling requirements, particularly for scavengers capable of tearing open carcass skin, such as vultures [14]. Vultures are highly effective foragers due to two key adaptations. First, they possess exceptional long-distance soaring abilities, enabling them to cover vast areas in search of food with a minimal energy expenditure [13,15]. Second, they are adapted to gather social information from conspecifics [16] and other scavenger species [17–19], which facilitates efficient resource discovery. These adaptations allow vultures to access carrion resources from great distances and tolerate high levels of competition and starvation [20,21]. In turn, the high efficiency of vulture scavenging has a significant impact on terrestrial ecosystems. The presence of vultures can dictate the rate at which carcasses are discovered and consumed, affecting ecosystem processes, such as nutrient cycling and trophic dynamics [22–26].

Given these unique characteristics, the vulture-carrion system serves as an ideal model to explore the determinants and outcomes of the Optimal Foraging Theory’s searching phase, considering both the consumer and the resource perspectives. In this study, we conducted a field experiment to examine the determinants of vulture foraging and its impact on ungulate carcass consumption. For this purpose, we integrated GPS tracking of griffon vultures (*Gyps fulvus*) with camera-trap monitoring of livestock carcasses in two Iberian regions with contrasting carrion availability. On the one hand, from the consumer perspective, we analysed how various factors, such as distance to the resource, hunger level and competition, influenced the vultures’ decisions to descend to experimental carcasses. On the other hand, from the resource perspective, our monitoring of these carcasses provided insight into how the vultures’ individual decisions shaped carrion consumption patterns (e.g. time of carcass discovery and consumption).

Our analysis was framed within two competing hypotheses. The first hypothesis, *satiation hypothesis*, suggests that a vulture’s decision to descend to a carcass is primarily driven by its hunger level. Under this hypothesis, we predicted that: (i) vultures experiencing high hunger levels would descend to a carcass irrespective of their distance from it, carrion availability or the intraspecific competition (hereafter, competition) they might face (consumer perspective); and (ii) carcasses in regions with high resource availability would take longer to be visited and consumed, as vultures in those areas would be more satiated (resource perspective). This hypothesis aligns with vultures exhibiting a type-II or logarithmic functional response, whereby, beyond a certain point, increases in resource density no longer result in higher consumption rates [4]. The second hypothesis, *opportunity hypothesis*, posits that vultures will descend to a carcass whenever an opportunity arises, regardless of their hunger level, carrion availability or competition, due to the ephemeral and unpredictable nature of carrion. Under this hypothesis, we expected that: (i) vultures would descend to a carcass if they were near enough to the carcass, indicating that distance to carcass is the primary determinant of their foraging behaviour (consumer perspective); and (ii) there would be minimal differences in carcass detection and consumption times between areas with varying carrion availability, as vultures would tend to exploit every feeding opportunity (resource perspective). Under this hypothesis, vultures would exhibit a type-I or lineal functional response, i.e. with little to no satiation [4].

## 2. Methods

### 2.1. Study areas

**Figure 1.**
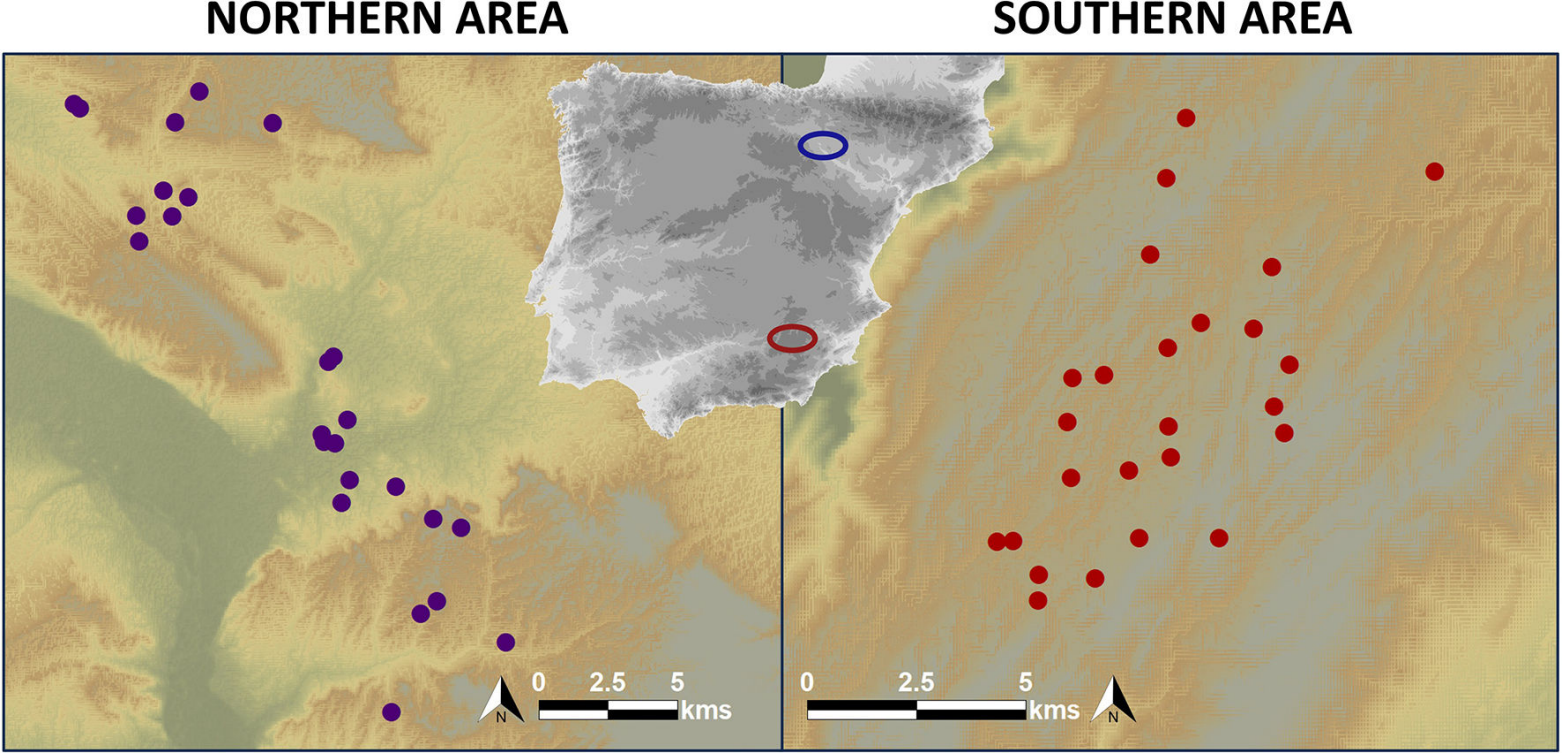
Location of the study areas in northern (blue ellipse) and southern (red ellipse) Spain. Dots show the distribution of experimental carcasses. Carcasses do not have the same density in both areas, therefore different scales have been used for a better graphic representation.

We GPS-tracked vultures and deployed experimental carcasses in two distinct Spanish Mediterranean regions: the Bardenas Reales de Navarra Natural Park in northern Spain (hereafter, northern area), and the Sierras de Cazorla, Segura y las Villas Natural Park in southern Spain (hereafter, southern area). The northern area is characterized by flat terrain, with elevations ranging from 280 to 659 m a.s.l. and an annual average temperature of 14.5°C. It is primarily composed of cereal steppes, surrounded by extensive irrigated farmlands [27,28]; figure 1). In contrast, the southern area encompasses a karstic plateau within a mountainous ridge predominantly covered by pine forests [29]. The elevation in this region spans from 500 to 2107 m a.s.l., with an annual average temperature of 13.8°C.

Both regions support significant vulture colonies, though precise population estimates at the study area scale are not available [30]. Carrion is abundant in both regions, but the northern area has notably higher ungulate carrion availability. Specifically, the estimated carrion production within a 50 km radius of the centre of the northern study area is 2668.33 ± 3847.97 kg year^−1 ^km^−2^, whereas in the southern study area it is 342.24 ± 40.51 kg year^−1 ^km^−2^ [31]. In the northern region, the primary source of carrion comes from intensive pig farming, with secondary contributions from sheep farming [32]. The southern area relies more on extensive livestock farming, with significant contributions from transhumant herds during the summer months [33].

### 2.2. Vulture trapping and tagging

We used cannon nets baited with sheep carcasses to trap 30 griffon vultures in the southern area from December 2014 to January 2015. Similarly, between December 2015 and April 2016, 31 griffon vultures were trapped in the northern area using the same method. All captured individuals nested or roosted within 100 km of the trapping site [34]. During handling, we identified all individuals as adults, with ages exceeding 7 years, based on feather patterns and other morphological traits [35]. To determine their sex, we used molecular techniques [36], resulting in a total of 15 males and 16 females in the northern population, and 19 males and 11 females in the southern population.

Each vulture was equipped with a 90 g GPS device (https://e-obs.de/) attached with a backpack harness [37]. Device settings varied during the study to record as many locations as possible, depending on weather conditions and battery status (details in electronic supplementary material, table S1). All individuals were monitored from the time of tagging until the end of 2018, except those that died or whose devices failed prematurely [38].

### 2.3. Carcasses monitoring

We monitored 24 livestock carcasses (23 sheep and one goat) in July 2015 within the pastureland area of the southern region and 25 sheep carcasses in July 2016 in the northern area (figure 1). Carcasses were placed near the vulture-trapping sites, always in open spaces to ensure they were fully visible from the air [39]. Simultaneous carcasses (never exceeding two carcasses with soft tissues at a time) were separated by more than 1.5 km from each other [39]. All carcasses were secured to the ground and placed before 11:00 to align with the vultures’ activity patterns [39].

We used camera traps (Bushnell NatureView Cam HD Max) to monitor each carcass [40]. The cameras were positioned at 5−20 m from the carcasses to capture clear images of vulture activity. These cameras were scheduled to operate from 6:30 to 21:30 to encompass most of the vultures' daily activity period [41]. The recording frequency was one photo per minute and for each photo, we recorded the time and the number of vultures present.

### 2.4. Consumer’s foraging decisions: defining the response and explanatory variables

To analyse the behaviour of tracked vultures in relation to experimental carcasses, we defined a binomial response variable called *Down*. This variable indicates whether each tracked individual descended to each of the experimental carcasses (yes/no). To determine whether a vulture descended to a carcass, we established a 50 m radius buffer around each carcass. Based on our field experience, this distance includes the range within which a vulture could perch before or after accessing the carcass. A vulture was considered to have descended if it met the following criteria: (i) we recorded at least one GPS location of the vulture within the 50 m buffer; (ii) the altitude above ground of this location was c. 0, calculated using a 30 × 30 m resolution Digital Elevation Model; (iii) the vulture’s ground speed was less than 3 m s^−1^, indicating that it was perched or moving slowly (walking). For this calculation, we included all tracked individuals, regardless of their distance from the carcass, because social attraction can (theoretically) trigger a cascading effect among foraging vultures, leading to long-distance contagious chains that may extend for hundreds of kilometres [16].

Then, to understand the factors influencing the probability of a vulture descending to a carcass, we calculated seven explanatory variables. First, we estimated the distance between each GPS-tracked vulture and the carcass at the moment of carcass discovery, which we defined as the time when the first photograph showing a vulture on the carcass was obtained. From this distance, we derived three explanatory variables: (i) *HorizontalDist*: the linear horizontal distance between the GPS position of the vulture and the carcass; (ii) *VerticalDist*: the altitude of the vulture above the ground; (iii) *RealDist*: the Euclidean distance, considering both horizontal and vertical components, between the GPS position and the carcass (see electronic supplementary material, figure S1 for a graphical representation of the variables related to the distance between vultures and carcasses). To evaluate the impact of hunger on the decision to descend, we used accelerometer data from the GPS devices to estimate the time of the last meal for each vulture (see [38] and [42] for methodological details). From this information, we derived two additional explanatory variables: (iv) *LastMeal*: the time in hours since the vulture’s last recorded meal; (v) *NoofMeals*: the number of times the vulture ate during the preceding week. To assess the effect of intraspecific competition, we introduced the variable (vi) *Competition:* the number of vultures detected at each carcass when a GPS-tracked individual was within 4 km of the carcass. This distance is the upper limit from which vultures can start descending towards a carcass based on previous bibliographic data [12]. Therefore, we assumed that at this distance the vultures are able to efficiently assess the number of intraspecific competitors around the carcass. Finally, to measure the effect of how long the carcass was available, we included the variable (vii) *Duration*: the time elapsed from the first photograph of a vulture at a carcass to the point when the carcass was completely consumed, leaving only bones and skin [40].

### 2.5. Consumer’s foraging decisions: principal component analysis

To address the overlap among several explanatory variables representing similar factors, such as distance or hunger, we conducted a principal component analysis (PCA) to identify the most relevant variables for describing the entire dataset. The use of PCA and a subsequent K-means clustering was chosen as we aimed to explore the underlying structure of the data rather than test a direct hypothesis about individual predictor effects (as could be done with a Logistic Regression model).

First, we normalized all the variables to mitigate the impact of varying scales among them. We excluded sex and study area from the PCA because they are two-level factor variables, which tend to stratify the results, particularly when dealing with smaller sample sizes [43]. Following the normalization, we computed the PCA to obtain the principal components, including coefficient data, score data and latent data. Once the principal components were generated, we extracted the latent data to calculate the total variance explained by each component [43]. We then computed the percentage of variance explained by each principal component, providing a relative measure of each component’s importance in explaining the data. To determine which variables contributed the most to the total variance in the dataset, we used the variance weight indicator, derived from the explained variance percentage [43]. The PCA analyses were conducted using MatLAB [44].

### 2.6. Consumer’s foraging decisions: K-means clustering

We used K-means to analyse the influence of different variables on the vultures’ decision of descending or not to a carcass. K-means clustering is particularly useful for our study case for several reasons: first, it can help to identify patterns in the data, even for small datasets; second, this method allows us to visually explore the results and understand the broad relationships between datapoints; third, clustering can be used to identify the key features that some particular datapoints have in common [45]. We used the elbow method [46] to determine the optimal number of clusters to be created, which were three in our case. After that, we implemented the clustering using variables PC1, PC2 and PC3 of our PCA (see §3). Then, (i) the analysis identifies the cluster centroids, and (ii) each observation in the feature space is assigned to its nearest centroid (based on its Euclidean distance from the centroid). Moreover, (iii) the process assigns a new centroid by calculating the mean of the observations clustered together in step ii. Steps ii and iii are repeated until convergence, which occurs when, for a fixed number of iterations, the positions of the centroids in the clusters do not change. K-means outputs the cluster to which each observation belongs, allowing a good visualization of the different trends in the data, i.e. different vulture behaviours. These analyses were performed using MatLAB [44].

### 2.7. Resource use patterns

To investigate carrion consumption patterns in both study areas, we defined and compared between areas the following four variables: (i) *T. discovery*: time since the carcass was placed until the first picture of a vulture; (ii) *T. maximum*: time since the first picture of a vulture until the maximum number of vultures counted in a picture was reached; (iii) *T. depletion*: time since the first picture of a vulture until the carcass was completely consumed; and (iv) *Max*: the maximum number of vultures counted in a carcass. Carcasses that were not entirely consumed by the end of the study or moved out of camera range (due to scavengers displacing them) were excluded from this analysis. Specifically, we excluded two carcasses from the northern area, one because it was not fully consumed and the other because it was displaced. We compared these variables between the two study areas through Mann–Whitney *U* test in R [47].

## 3. Results

### 3.1. Consumer’s foraging decisions

We obtained 19 cases in which at least one of the GPS-tracked vultures descended to an experimental carcass. More specifically, these cases involved 15 different carcasses (five in the northern area and 10 in the southern area) and 12 different individuals (five in the northern area and seven in the southern area; table 1 and electronic supplementary material, figure S2). At the moment that the first vulture appeared (i.e. recorded by the camera) in each monitored carcass, the vultures that descended were located at a median horizontal distance of 3762 m (625–22 606 m). Furthermore, the distance from which vultures initiated their descent to the carcass had a median of 2200.76 m (358.8−7062.7 m; see more details in table 1).

**Table 1.** Status of each GPS-tracked vulture in two Spanish areas that descended to the experimental livestock carcasses. *DescDist* represents the distance at which the vulture began its descent. *HorizontalDist*, *VerticalDist* and *RealDist* represent the straight-line distance from the vulture to the carcass, the altitude of the vulture above the ground, and the Euclidean distance between the vulture and the carcass, respectively. All distances are in m. *LastMeal* indicates the hours since the vulture’s last meal. *NoofMeals* shows the number of times the vulture ate in the preceding week. *Competition* is the number of vultures present at the carcass when the GPS-tracked vulture approached within 4 km (see §2). Finally, *Duration* indicates how long it took for each carcass to be consumed after at least one vulture descended.

individual ID	carcass ID	study area	*descdist*	*horizontaldist*	*verticaldist*	*realdist*	*lastmeal*	*No of meals*	*competition*	*duration*
T1L	car46	northern	2370.0	5387	71	5390	21.95	2	0	3.22
T21	car38	northern	546.2	4984	79	4988	14.12	7	2	4.93
T21	car39	northern	1032.0	13 470	45	13 471	0.37	8	0	23.92
T24	car29	northern	3142.6	9033	210	9033	18.20	2	1	6.73
T2H	car33	northern	746.6	710	144	714	59.20	1	28	9.58
T33	car33	northern	454.9	623	193	627	20.68	5	8	9.58
L8J	car11	southern	3002.0	2993	300	3001	17.65	2	0	3.00
T08	car8	southern	7062.7	9173	125	9175	18.73	4	1	6.98
T0C	car20	southern	4087.6	22 587	0	22 589	21.85	4	1	9.27
T0L	car12	southern	1286.3	7989	201	7990	16.68	3	7	5.93
T0X	car1	southern	1281.5	1283	0	1294	135.57	1	0	5.05
T11	car3	southern	2029.1	3085	130	3092	16.00	4	0	6.28
T11	car12	southern	2429.7	2420	87	2428	36.85	1	0	5.93
T11	car13	southern	358.8	6062	115	6232	37.38	3	0	12.50
T11	car16	southern	3754.9	3796	211	3797	38.10	2	0	6.80
T11	car22	southern	2067.14	2072	169	2075	16.53	4	0	4.67
T1J	car3	southern	3324.1	8442	215	8447	11.95	5	5	6.28
T1J	car12	southern	1799.7	1803	0	1828	18.35	9	0	5.93
T1J	car15	southern	1038.7	1041	0	1048	16.28	9	0	4.57

**Figure 2.**
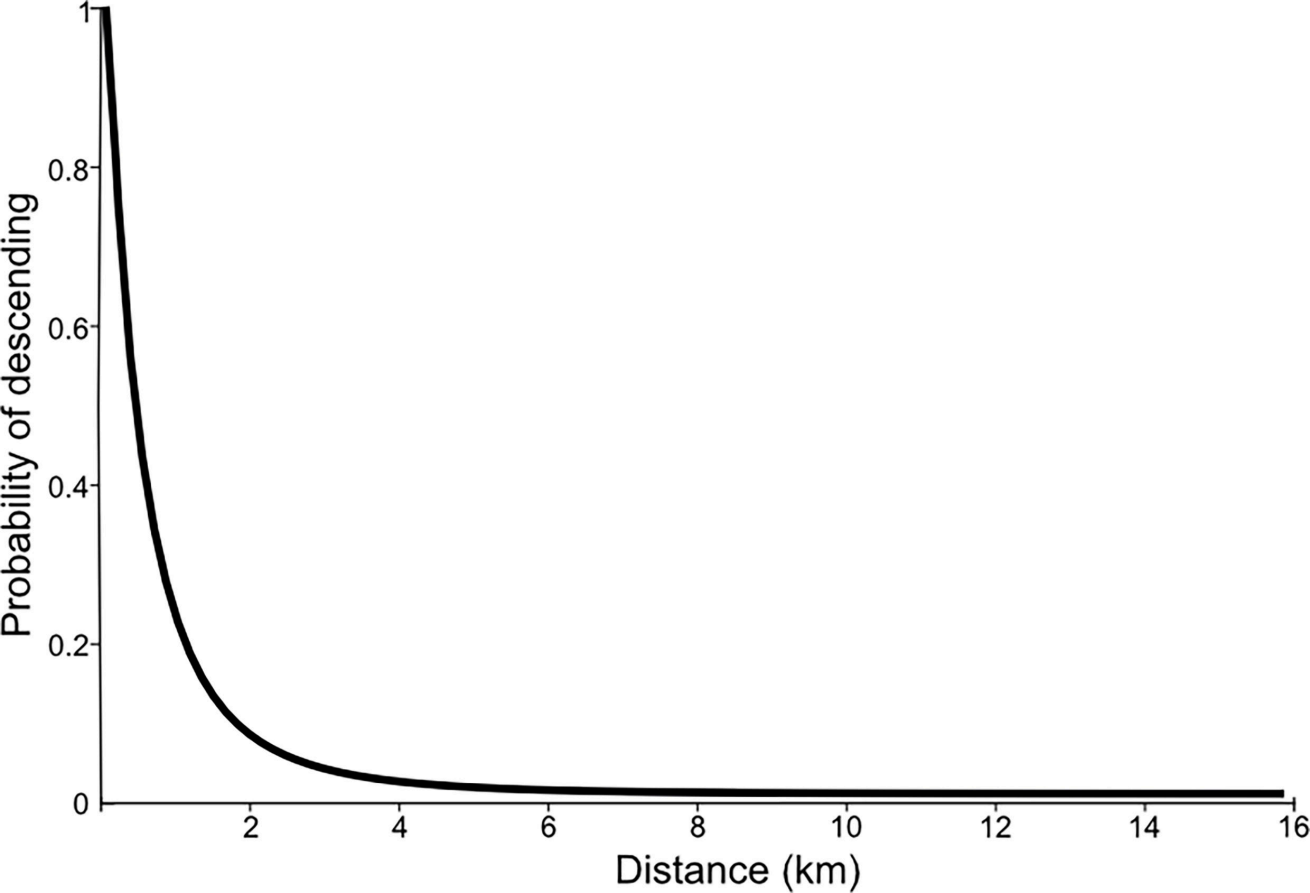
Probability that a vulture descends to a carcass, according to the distance between them. Our analysis focuses on estimating the probability that a vulture descends to a carcass based on its distance to it at the moment of descent (using only the cases when a vulture actually descended (see electronic supplementary material, figure S3)), rather than on the raw frequencies of vultures initially positioned at different distances. To achieve this, we used a Kernel Density Estimation (KDE). From this function, we derived the Cumulative Distribution Function (F) and subsequently calculated the complementary Cumulative Distribution Function (1F), which is displayed in this figure.

The PCA showed that PC1, PC2 and PC3 explained 69% of the variance across the dataset (table 2). PC1 mainly contained information about the distance between vultures and carcasses (main variables: *HorizontalDist* and *RealDist*), and explained 31% of the variance; PC2 was related to vultures’ hunger level (main variables: *NoofMeals* and *LastMeal*), and explained 23% of the variance; and PC3 represented a mix between intraspecific competition and the duration of the carcass (main variables: *Duration* and *Competition*), and explained 15% of the variance (table 2). Thus, the process of deciding to descend on a carcass seemed to be influenced primarily by the distance at which the individual was located, secondarily by its hunger level and, to a lesser extent, by the time it took for the carcass to be consumed and possible intraspecific competition. The probability of a vulture descending on a carcass decreased as a function of the distance from the carcass (at the moment of the first picture of a vulture at the carcass) and dropped sharply after 1 km (figure 2).

**Table 2.** Results of the PCA to relate several variables potentially explaining the probability for a vulture to descend on a carcass. The table shows the coefficients for each variable in the linear combinations that form each principal component. The last row indicates the variance explained by each principal component.

variables	PC1	PC2	PC3	PC4	PC5	PC6	PC7
horizontaldist	0.64	−0.18	−0.08	0.16	0.14	0.02	0.71
verticaldist	−0.36	−0.15	−0.16	0.53	0.73	0.08	0
realdist	0.64	−0.18	−0.08	0.16	0.14	0.02	−0.71
lastmeal	−0.1	−0.6	0.35	−0.19	−0.03	0.68	0
No of meals	0.11	0.65	−0.19	0.11	−0.02	0.72	0
competition	−0.14	−0.33	−0.57	0.44	−0.59	0.08	0
duration	0.02	0.14	0.69	0.66	−0.27	−0.06	0
explained variance	31%	23%	15%	14%	10%	7%	0%

**Figure 3.**
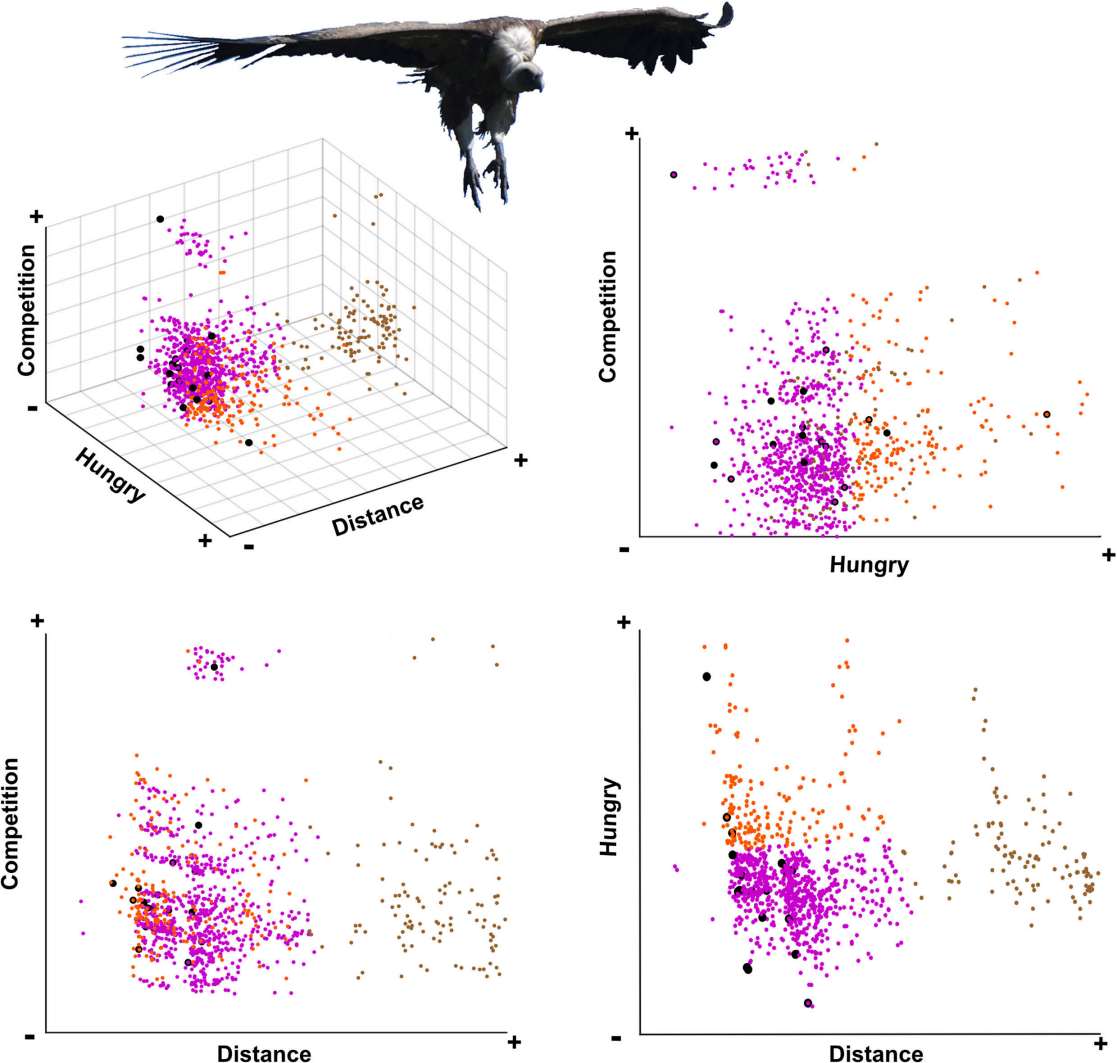
Factors affecting the probability that a vulture descends to a carcass. (A) Three-dimensional graphical representation of the K-means clustering analysis using the three most relevant principal components (PCs) from the normalized PCA (see §3). Each point represents the status of each GPS-tracked vulture in relation to each monitored carcass. Black dots indicate instances where the monitored vultures descended to the carcass. The other colours represent the three clusters identified by the analysis. (B–D) display the same results in a two-dimensional space, comparing the three variables pairwise.

K-means clustering was performed using the three main components (PC1, PC2 and PC3 renamed as distance, hungry and competition axis) selected from the PCA output and resulted in three clusters (figure 3). The first cluster (brown dots in figure 3) included events in which the monitored vultures were away from the carcass when the first vulture arrived to it. The second cluster (orange dots in figure 3) represented events where the monitored vultures were close to the carcass and had hunger values above the mean. The third cluster (purple dots in figure 3) grouped events where the monitored vultures were close to the carcass but had hunger values below the mean. Thus, vultures that descended to the carcasses (black dots in figure 3) were generally those that (i) were close to the carcass (see figure 3) and (ii) had intermediate-low hunger values. Additionally, these cases occurred in carcasses with low-competition values (PC3; see table 1).

### 3.2. Resource use patterns

**Figure 4.**
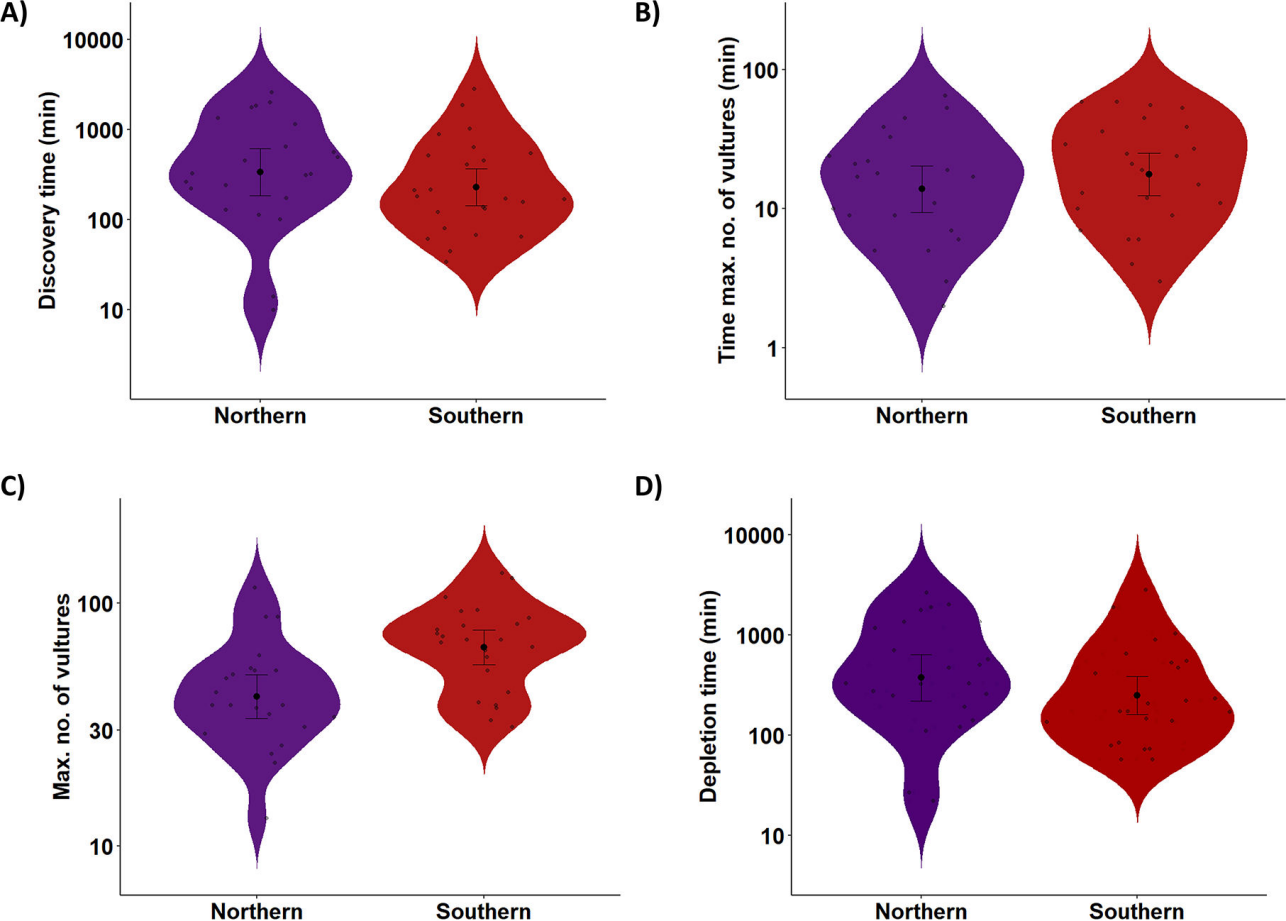
Comparison of carrion consumption patterns in the two study areas. (A) The time taken to discover each carcass, and (B) the time at which the highest number of vultures was recorded. (C and D) Compare the duration of consumption (from the arrival of the first vulture until the carrion is fully consumed) and the maximum number of vultures recorded at each carcass, respectively.

Carcass consumption patterns were very similar between areas (electronic supplementary material, table S2, figure 4). Accordingly, we did not find significant differences in *T. discovery* (W = 327, *p* = 0.282), *T. maximum* (W = 244, *p* = 0.502) and *T. depletion* (W = 344, *p* = 0.151). We only found significant differences in the maximum number of vultures detected per carcass (*Max*; W = 138, *p* = 0.003), which was higher in the southern population (figure 4).

## 4. Discussion

Our results support the *opportunity hypothesis*, whereby vultures are more likely to descend on a carcass if they are close to it, with relatively lower importance of other factors such as hunger and potential competition. Therefore, it seems that vultures, facing the unpredictability of their trophic resource, try to eat whenever a nearby opportunity arises. The absence of satiation indicates that, in our study system, vultures follow a type-I functional response towards carrion resources [4]. This is in agreement with the fact that vultures in the northern study area, which has higher carrion availability [31], tend to have greater body mass (authors unpublished data), as they eat even with relatively little hunger according to our proxies based on accelerometry data. According to our prediction, the consequence for carrion consumption patterns is that the times of carcass discovery and depletion, as well as the time needed to gather the maximum number of consumers, is similar in scenarios with different resource availabilities.

### 4.1. Consumer’s foraging decisions

Distance to carcass was the main factor determining the vultures’ decisions of descending or not to a carcass. Vultures that descended to any of the experimental carcasses were close to them when the first conspecific arrived. At this distance, vultures perhaps can assess whether it is worthwhile to reach the carcass or not. Given the low importance of the competition variable in our PCA, vultures likely rely on social information, such as the number of vultures flying over the area, rather than the number of vultures actually present around the carcass. *A priori*, this would also apply to interspecific social information, with the activity of other flying scavengers (e.g. eagles, kites, corvids) in the air likely to be more important than their presence around the carcass itself [19]. Although these factors could not be accounted for in our experiment, it is unlikely that they affected our results given the relatively high density of vultures in both study areas. However, in situations of defaunation, the number of vultures or other scavenger species in flight could become a determining factor. The disappearance or reduction of conspecific or interspecific individuals could disrupt the aerial information transmission network, potentially altering the functional response of the vultures (see below).

How vultures use social information to find carcasses has concerned ecologists for decades [17,18,48,49]. Cortés-Avizanda *et al*. proposed two alternative models to explain how this information is transmitted [16]. First, information about carcass location could be transmitted when uninformed vultures follow those that know where the carcass is, forming a theoretically infinite chain of contagion. Second, vultures could forage in a ‘searcher and scrounger’ scenario, whereby individuals locate the carcass by themselves or by following others who are more informed, but always within a finite spatial buffer around the carcass. The significance of proximity to the carcass in our findings appears to support the second hypothesis. Moreover, considering that individuals descending to a carcass were within a distance less than 23 km, we can infer that the carcass’s influence extends to approximately a 20 km-radius. Nonetheless, the attractiveness of the carcass diminishes sharply with increasing distance, being almost marginal beyond 1 km (see figure 2). This may be due to the attraction buffer reaching several tens of kilometres, but the vultures need to be much closer to correctly assess the relationship between competitors (flying or perched) above or around the carcass and the amount of carrion remaining. Supporting this hypothesis, in our experiment, all vultures except one commenced their descent from distances of 4.5 km or less (see table 1), coinciding with findings in other *Gyps* species [12].

### 4.2. Resource use patterns

Differences in carrion availability in the two study areas did not result in different discovery times, which could indicate the relation between the number of consumers and the available resources is similar in both areas, and/or that vultures in the area with relatively higher carrion availability eat more. We also found no differences in the time when the maximum number of vultures is reached and in the time the carcass is completely consumed. Within a type-I functional response, these two variables would represent the time at which the consumer physically cannot deplete resources at a higher rate and the time at which consumers abandon the resource in search of another [4]. The lack of differences in these variables indicates that the functional response is similar in both populations.

Given that variations in carcass availability did not result in differences in other variables related to the consumption by vultures, it is unlikely that this factor accounts for the larger number of vultures gathering at southern carcasses (figure 4). It is well known that vultures coming from other populations, either nearby or distant, can congregate in areas with abundant food [34]. Also, previous studies have shown that the presence of transhumant livestock herds during the summer (coinciding with our study period) increases vulture density in our southern study area [33]. Thus, the difference in the maximum number of vultures per carcass could be attributed to a higher total number of foraging vultures in the southern study area compared with the northern study area.

### 4.3. Final remarks

The type-I functional response is uncommon in nature [4] and is traditionally restricted to filter-feeding organisms [50]. However, our results, added to the fact that vultures virtually lack a handling phase, suggest that vultures present a type-I functional response, at least in scenarios of abundant medium-sized ungulate carrion and large vulture populations. Vultures are likely to have evolved this type of functional response due to the spatiotemporal unpredictability of carrion, forcing them to exploit every available feeding opportunity. However, this life strategy is likely not rigid, and vultures could transition to a type II functional response when the ratio of vultures to food availability was very low. These scenarios should be mostly restricted to events that generate large quantities of carrion, such as mass mortalities of ungulates [51] and megaherbivore carcasses [40], as well as to vulture population declines. In such circumstances, vultures can fully satiate their hunger without the need to constantly switch between resource patches, as typically observed in type I responses. In the present context of global environmental change, where humans are the primary producers of carrion in many regions and most vulture populations are below the carrying capacity due to anthropogenic causes, vultures will be increasingly prone to show a type II functional response. An obvious expected result is the reduction in the rate of carrion recycling [22–25], which could lead to wide impacts on ecosystem functioning and ecosystem services [52].

The OFT is a well-established theoretical framework. However, our findings demonstrate that there are still important avenues that require further empirical attention. The unique vulture-carrion system facilitates a comprehensive examination of OFT from the standpoint of both the consumer and the resource. Our results provide insights into the relative importance of intrinsic (e.g. hunger) and extrinsic (e.g. distance, competition) factors in the foraging behaviour of vultures and how these behaviours shape carrion consumption patterns in contrasting environments. Also, our findings suggest that the importance of type I functional response in nature in general, and in vertebrate communities in particular, may have been underestimated. Identifying study models with extreme foraging strategies may help to expand our knowledge of the evolution of different OFT components, as well as to understand the consequences for ecosystem functioning.

## Data Availability

The data are uploaded as supplementary material [53].
